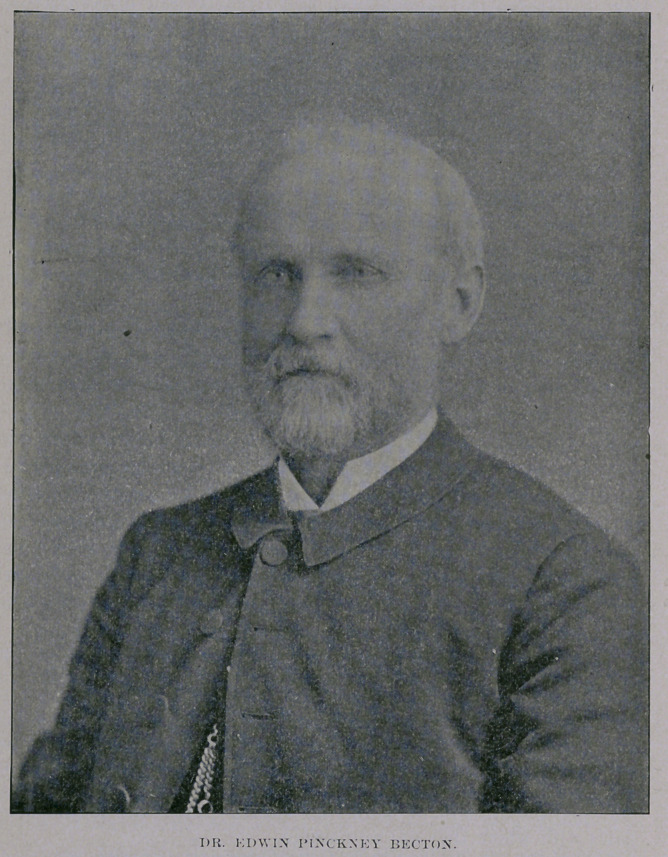# Dr. Edwin Pinckney Becton

**Published:** 1901-01

**Authors:** 


DR. EDWIN PINCKNEY BECTON.

THE

### TEXAS MEDICAL JOURNAL.

AUSTIN, TEXAS.

A MONTHLY JOURNAL OF MEDICINE AND SURGERY.

EDITED AND PUBLISHED BY

F. E. DANIEL, M. D., AND S. E. HUDSON, M. D.

Published Monthly at Austin, Texas, by Drs. Daniel and Hudson. Subscription price $1.00 a year in advance.

Eastern Representative: John Guy Monihan, St. Paul Building, 220 Broadway, New York City.

Official organ of the West Texas Medical Association, the Houston District Medical Association, the Austin District Medical Society, the Brazos Valley Medical Association, the Galveston County Medical Society, and several others.

### Dr. Edwin Pinckney Becton.

THE able, distinguished and greatly loved Texas physician, Dr. E. P. Becton, died in Austin January 14th inst.

When the news of his death was flashed over the wires, it carried grief and sorrow to many loving hearts throughout the State. It was, too, a great shock to those who knew him best, and who had recently seen him in the vigor of a mature and well preserved manhood. Altho' he was in his 66th year, he was strong and vigorous, and in the full possession and enjoyment of all his faculties, mental and physical. His friends believed and hoped that he had many more years of life and usefulness before him. Dr. Becton was not only one of the ablest and most gifted and brilliant men in the medical profession of the State, universally admired and beloved for his many excellencies of head and heart, but he was one of the land marks, one of the "fathers," one of the old Confederate soldier-surgeons. For many years he has been one of the stars in the medical profession, and as one of its wisest counselors and most energetic workers in the State Medical Association, of which he was president in 1886, he will be sadly missed and mourned. He was a leader. When there was to be a telling speech made, Becton was looked to spontaneously by the whole body to make it, and he was always ready, always willing, and always spoke just the right thing at the right time—carrying all before him, his matchless rhetoric and splendid delivery eliciting the warmest admiration and applause. He was doubtless the most gifted and brilliant extemporaneous speaker we had—excelled by none —and equalled perhaps alone by the peerless Swearingen, whose loss we so lately mourned, and whose splendid presence and thrilling eloquence we still miss at our annual reunions. Peace to his soul.

Dr. Becton was a genial and most lovable man. Aside from his high and beautiful character as a physician, he possessed the qualities of head and heart that won the respect, admiration and esteem of all with whom he was thrown. Genial and gentle, earnest and energetic—he entered into all the duties and responsibilities of life—professional, social, business—with the zest of an enthusiast. He was optimistic. He was a staunch Christian and died in the faith of a glorious immortality. At the time of his death he was superintendent of the State Institution for the Blind, a position to which he was invited by Governor Sayers two years ago and to which he had just been re-appointed by the Governor upon entering on his second term. The doctor had greatly endeared himself to the unfortunates and the staff of the Institution by his gentle, sympathetic manners, by his able administration and by his watchful care for the health, happiness and welfare of the State's unfortunate wards entrusted to his care. A long farewell to our beloved colleague: the brave and brilliant Becton!

Biographical.—Dr. Edwin Pinckney Becton was born in Gibson county, Tenn., June 27, 1834. He came to Texas in 1841 with his parents, who settled at San Augustine, where he was early placed at school and acquired the rudiments of a good literary education. He chose the study of medicine and entered the office of Dr. A. R. Hamilton, at New Danville, Texas. In the winter of 1855-56 he attended lectures at Nashville, Tenn. At the close of the term he went to Murfreesboro, Tenn., and studied under the prominent physicians Jas. E. and Robert S. Wendell. The next year he entered the medical department of the University of Tennessee, graduating with high honors. He commenced practice at New Danville, Tex., the year of his graduation. Later he attended medical lectures at the University of Louisville, Ky., 1874; at University of Maryland, Baltimore, 1879-80; at Tulane University, La., 1886, and in 1891 at the Polyclinic, New York.

In 1862 he entered the Confederate army as a private soldier. He was appointed assistant surgeon of Fitzhugh's regiment, Mc-

Colloch's brigade, Walker's division, and he was soon thereafter recommended for promotion and assigned to duty with 22nd regiment of Texas infantry. This position he held till the close of the war, when he settled in Tarrant, in Hopkins county, and resumed the practice of his profession. In March, 1874, he moved to Sulphur Springs in the same county, where he resided until the year 1895, when he was appointed Superintendent of the State Institution for the Blind. He was a member of the 12th Texas Legislature.

## Figures and Tables

**Figure f1:**